# Transcranial magnetic stimulation and post-traumatic stress disorder

**DOI:** 10.1192/j.eurpsy.2022.1733

**Published:** 2022-09-01

**Authors:** N. Moura, A. Fraga, J. Facucho-Oliveira, F. Azevedo, C. Laginhas, D. Esteves-Sousa

**Affiliations:** 1 Centro Hospitalar Lisboa Ocidental, Psychiatry, Lisbon, Portugal; 2 Hospital de Cascais, Psychiatry, Alcabideche, Portugal; 3 Centro Hospitalar de Lisboa Ocidental, Psychiatry, Lisbon, Portugal

**Keywords:** PTSD, TMS

## Abstract

**Introduction:**

Post-traumatic stress disorder (PTSD) is a psychiatric disorder characterized by symptoms from four clusters after exposure to a traumatic event: re-experiencing symptoms including flashbacks and nightmares, hyperarousal, avoidance of internal and external stimuli related to trauma, and negative alterations in mood and cognition. As a noninvasive intervention that uses induction of electromagnetic fields to modulate cortical circuitry, TMS has a substantial body of literature demonstrating safety, tolerability, and efficacy in depression and potentially PTSD.

**Objectives:**

Our aim is to perform a non-systematic review of the literature regarding TMS and PTSD

**Methods:**

A semi-structured review was conducted on Pubmed concerning TMS and PTSD

**Results:**

The majority of studies utilize repetitive TMS targeted to the right dorsolateral prefrontal cortex (DLPFC) at low frequency (1 Hz) or high frequency (10 or 20 Hz), however others have used alternative frequencies, targeted other regions, or trialed different stimulation protocols utilizing newer TMS modalities such as theta-burst TMS (TBS). It is encouraging that were positive outcomes have been shown, and often sustained for up to -3 months, nevertheless there is a paucity of long-term studies directly comparing available approaches.

**Conclusions:**

TMS appears safe and effective for PTSD, although important steps are needed to operationalize optimal approaches for patients.

**Disclosure:**

No significant relationships.

